# Bacterial community structure and diversity of common mosquito species in Chengdu: Insights from PacBio third-generation sequencing and public health implications

**DOI:** 10.1371/journal.pntd.0013177

**Published:** 2026-02-12

**Authors:** Yifan Xing, Rong Lu, Wenjia Tian, Zelin Li, Wei Zhang, Kai Xu, Liangli Deng, Shuangfeng Fan

**Affiliations:** 1 Chengdu Center for Disease Control and Prevention (Chengdu Institute of Health Supervision), Chengdu, China; 2 Institute of Zoology, Chinese Academy of Sciences, Beijing, China; Egerton University, KENYA

## Abstract

Mosquitoes, as critical vectors of diseases such as Japanese encephalitis, dengue fever, and yellow fever, pose significant public health risks in Chengdu, a subtropical city in southwestern China. The present study ecological surveillance and PacBio third-generation sequencing to characterize the symbiotic microbiota of four dominant mosquito species (*Aedes albopictus*, *Culex pipiens quinquefasciatus*, *Culex tritaeniorhynchus*, and *Armigeres subalbatus*) across urban and rural habitats. From 2020 to 2024, mosquito density monitoring revealed spatial heterogeneity(*Aedes albopictus*, *Culex pipiens quinquefasciatus*, *Culex tritaeniorhynchus*, and *Anopheles sinensis*), with outer ring areas exhibiting the highest density (34.69 mosquitoes per trap per night), while central urban zones had the lowest (3.60). Sequencing identified 717 high-quality Amplicon Sequence Variants (ASVs), with *Aedes albopictus* harboring the most unique bacterial species (191). Beta diversity analysis demonstrated distinct microbial clustering among species, driven by *Pseudomonadota* dominance (54.27–93.89%) and variations in secondary phyla (*Bacteroidota, Campylobacterota*). Functional prediction analysis via the Kyoto Encyclopedia of Genes and Genomes (KEGG) revealed significant disparities in the abundance of human disease-associated pathways across mosquito symbiotic microbiota (*P* = 0.049), with the disparities primarily observed in pathways related to bacterial, viral, and parasitic infections—categories of substantial public health relevance. Notably, *Wolbachia (clade B)* and *Klebsiella variicola* exhibited species-specific abundance patterns, underscoring their respective roles in potential pathogen suppression and public health risks. Unclassified taxa (*norank_d__Bacteria, norank_p__Candidatus_Hydrogenedentes*) clustered near novel mosquito-associated spirochetes, suggesting underexplored functional microbiota. This study establishes a foundational dataset for understanding mosquito-microbe interactions and inform the development of targeted strategies for mitigating vector-borne disease.

## Author summary

Mosquito-borne diseases like dengue and Japanese encephalitis disproportionately affect subtropical regions. Understanding how mosquitoes’ native microbes influence disease transmission remains a critical challenge, especially in under-resourced areas. Chengdu, a megacity in southwestern China, faces unique challenges due to rapid urbanization, climatic suitability for mosquito proliferation, and disparities in public health infrastructure compared to eastern coastal regions. This study integrates five years of ecological surveillance (2020–2024) and PacBio third-generation sequencing to map mosquito density patterns and characterize symbiotic bacterial communities in four dominant mosquito species (*Aedes albopictus*, *Culex pipiens quinquefasciatus*, *Culex tritaeniorhynchus*, and *Armigeres subalbatus*). Key findings reveal stark spatial heterogeneity in mosquito density, with rural outer-ring areas harboring 10-fold higher densities (34.69 mosquitoes per trap per night) than urban centers. Notably, *Aedes albopictus* exhibited the highest diversity of unique symbiotic bacteria (191 species), including *Wolbachia (clade B)*, known to suppress arboviruses like dengue. Conversely, *Armigeres subalbatus* carried high abundances of *Klebsiella variicola*, an emerging human pathogen linked to severe infections. Functional annotation based on PICRUSt2 revealed significant species-specific variations in disease-related metabolic pathways within the mosquito symbiotic microbiota, with notable differences observed in the abundance of pathways associated with bacterial, viral, and parasitic infections. This work provides the first comprehensive baseline dataset on mosquito-microbe interactions in Chengdu, identifying actionable targets for biocontrol (e.g., leveraging *Wolbachia*) and facilitating the development of early-warning systems for pathogen surveillance. By bridging ecological, molecular, and public health perspectives, our findings offer critical insights for mitigating neglected tropical diseases in subtropical regions where socioeconomic disparities intersect with high disease burdens.

## Introduction

As a critical medical vector, mosquitoes transmit a spectrum of diseases, including Japanese encephalitis, dengue fever, and yellow fever, resulting in approximately 1 million deaths and over 700 million infections annually [1]. Chengdu, situated in the western Sichuan Basin, is characterized by a subtropical humid climate with concurrent rainy and hot seasons, short frost periods, and abundant green vegetation, creating an ideal habitat for mosquito proliferation. In recent years, ecological shifts, climate variability, and the hosting of large-scale international events have exacerbated the risks of emerging, re-emerging, and imported mosquito-borne diseases, imposing substantial pressure on local public health systems. Given the strong correlation between mosquito population distribution and disease transmission dynamics, there is an urgent need to investigate local mosquito density patterns to refine current prevention and control strategies.

Concurrently, advancements in research on insect-associated symbiotic microbiota have deepened our understanding of host-microbe interactions. Numerous studies demonstrate that symbiotic bacteria regulate critical physiological processes in insect hosts, including immune defense, reproductive modulation, and developmental pathways [2–5]. Specifically, certain mosquito symbionts influence vector competence for pathogen transmission [6,7], while gut microbiota enhance insecticide resistance by upregulating detoxification enzymes and stimulating metabolic pathways [8]. Elucidating and leveraging these symbiotic microorganisms for mosquito biocontrol represents an innovative frontier in combating vector-borne diseases.

With advancements in sequencing technologies, Academician Xu Jianguo proposed the “reverse pathogenomics” framework in 2019, a paradigm distinct from Koch’s postulates. This approach emphasizes proactive discovery of novel microorganisms in vectors via sequencing, followed by rapid assessment of their pathogenic potential and public health implications, enabling early deployment of targeted strategies to preempt emerging epidemics [9]. This methodology circumvents limitations inherent to traditional culture-based workflows (isolation, cultivation, and biochemical identification), which are often impractical for low-abundance or fastidious bacteria in small vectors like mosquitoes—particularly those requiring specialized conditions (e.g., anaerobic, low-temperature) and involving labor-intensive, time-consuming, and subjective biochemical analyses.

To address these challenges, The present study integrated mosquito light trap surveillance with third-generation high-throughput sequencing (PacBio) of the 16S rRNA gene. We conducted baseline monitoring of dominant wild mosquito species in Chengdu,to map their spatiotemporal density variations. Concurrently, we characterized the structure and diversity of their symbiotic microbiota. By identifying key microbial taxa and predicting their functional profiles, this work provides critical insights into the coevolutionary dynamics and interaction mechanisms between mosquitoes and their symbionts. Furthermore, it facilitates early detection of uncharacterized or exotic microorganisms, enabling the establishment of a pathogen surveillance framework. These efforts aim to inform proactive strategies for controlling endemic mosquito populations and mitigating risks posed by emerging, re-emerging, or unknown vector-borne diseases in Chengdu.

## Materials and methods

### Ecological monitoring of adult mosquitoes

From 2020 to 2024, adult mosquito surveillance was conducted in 22 districts of Chengdu (excluding Dongbu New Area) using the mosquito light trap method.In accordance with the the national surveillance protocol which specifically targets common mosquito species of public health importance in Chengdu, such as *Aedes albopictus*, *Culex tritaeniorhynchus*, *Culex pipiens quinquefasciatus*, *Aedes aegypti* and *Anopheles sinensis*. In urban areas, two sites each were selected from residential zones, parks, and hospitals. In rural areas, two sites each were chosen from residential houses and livestock sheds. With the exception of livestock sheds, all monitoring sites were situated in outdoor environments.Surveillance was performed twice monthly from April to November each year, with a minimum interval of 10 days between consecutive sessions. Two mosquito traps were deployed per habitat. The Traps were activated one hour before sunset and deactivated one hour after sunrise the following day. Captured mosquitoes were morphologically identified to species level based on characteristics such as the mesonotal scutum, tarsal segments, and color patterns, using standardized taxonomic keys.

### Sampling of mosquito specimens for symbiotic bacterial sequencing

Based on preliminary surveillance data, four national mosquito surveillance sites were selected in Dujiangyan City, Pengzhou City, Chongzhou City, and Qingyang District of Chengdu. From July to September 2024, live mosquito specimens were collected using human-baited traps, targeting only *Aedes albopictus*, *Culex* *tritaeniorhynchus*, *Culex pipiens quinquefasciatus*, and *Armigeres subalbatus*. Collected specimens were transported to the laboratory on the same day. Morphological identification of mosquito species was performed under stereomicroscopy according to taxonomic keys, focusing on diagnostic characteristics such as mesoscutum patterns, tarsal segment morphology, and pigmentation bands. Post-identification, blood-fed females were excluded from the sample pool. All confirmed specimens were cryopreserved at −80°C for subsequent molecular analyses.

### DNA extraction and PCR amplification

For each species (*Aedes albopictus*, *Culex pipiens quinquefasciatus*, *Culex tritaeniorhynchus*, and *Armigeres subalbatus*), 80 specimens were randomly selected and divided into 4 tubes (e.g., for *Aedes albopictus*, tubes were labeled A_a1 to A_a4), resulting in a total of 16 tubes. Each specimen was washed three times with 75% ethanol and phosphate-buffered saline (PBS) to remove surface contaminants, then blotted dry on autoclaved filter paper. Samples were transferred to sterile, nuclease-free grinding tubes, to which 500 μL of pre-cooled PBS was added. Homogenization was performed using a cryogenic grinding instrument at 90 Hz for 45 seconds.

Total microbial community DNA was extracted from the homogenized lysate using the FastPure Tissue DNA Isolation Kit according to the manufacturer’s instructions. The integrity of the extracted DNA was assessed by 1% agarose gel electrophoresis, and DNA quality was determined using a NanoDrop 2000 spectrophotometer.(Thermo Scientific, United States).The bacterial 16S rRNA genes were amplified using the universal bacterial primers 27F (5’-AGRGTTYGATYMTGGCTCAG-3’) and 1492R (5’-RGYTACCTTGTTACGACTT-3’). Primers were tailed with PacBio barcode sequences to distinguish each sample. Amplification reactions (20-μL volume) consisted of 2 × Pro Taq 10 μL, forward primer (5 μM) 0.8 μL, reverse primer (5 μM) 0.8 μL, template DNA(10 ng) and DNase-free water. The PCR amplification was performed as follows: initial denaturation at 95°C for 3 min; followed by 30 cycles of denaturing at 95°C for 30 s; annealing at 60 °C for 30 s and extension at 72°C for 45 s; and single extension at 72°C for 10 min;and end at 4°C (T100 Thermal Cycler PCR thermocycler, BIO-RAD, USA). After electrophoresis, the PCR products were purified using the AMPure PB beads (Pacific Biosciences, CA, USA) and quantified with Qubit 4.0 (Thermo Fisher Scientific, USA).

### DNA library construction and sequencing

Purified products were pooled in equimolar and DNA library was constructed using the SMRTbell prep kit 3.0 (Pacific Biosciences, CA, USA) according to PacBio’s instructions. Purified SMRTbell libraries were sequenced on the Pacbio Sequel IIe System (Pacific Biosciences, CA, USA) by Majorbio Bio-Pharm Technology Co. Ltd. (Shanghai, China). High-fidelity (HiFi) reads were obtained from the subreads, generated using circular consensus sequencing via SMRT Link v11.0.

### Data processing and statistical analysis

HiFi reads were barcode-identified and length-filtered. For bacterial 16S rRNA gene, sequences with a length < 1,000 or > 1,800 bp were removed. The optimized-HiFi reads were de-noised using DADA2 plugin in the Qiime2 [10] (version 2020.2) pipeline with recommended parameters, which obtains single nucleotide resolution based on error profiles within samples. DADA2 denoised sequences are usually called amplicon sequence variants (ASVs). To minimize the effects of sequencing depth on alpha and beta diversity measure, the number of sequences from each sample was rarefied to 4,090, which still yielded an average Good’s coverage of 99.97%. Taxonomic assignment of ASVs was performed using the Blast consensus taxonomy classifier implemented in Qiime2 and the SILVA 16S rRNA database (v138). The metagenomic function was predicted by PICRUSt2 (Phylogenetic Investigation of Communities by Reconstruction of Unobserved States) based on ASV representative sequences. PICRUSt2 [11] is a software containing a series of tools as follows: HMMER was used to aligns ASV representative sequences with reference sequences. EPA-NG and Gappa were used to put ASV representative sequences into a reference tree. The castor was used to normalize the 16S gene copies. MinPath was used to predict gene family profiles, and locate into the gene pathways. Entire analysis process was accord to protocols of PICRUSt2.

### Statistical analysis

Bioinformatic analysis of the mosquito symbiotic microbiota was carried out using the Majorbio Cloud platform (https://cloud.majorbio.com). Based on the ASVs information, rarefaction curves and alpha diversity indices including observed ASVs, Chao1 richness, Shannon index and Good’s coverage were calculated with Mothur v1.30.1 [12].The similarity among the microbial communities in different samples was determined by principal coordinate analysis (PCoA) based on Bray-curtis dissimilarity using Vegan v2.5-3 package. The PERMANOVA test was used to assess the percentage of variation explained by the treatment along with its statistical significance using Vegan v2.5-3 package. The linear discriminant analysis (LDA) effect size (LEfSe) (http://huttenhower.sph.harvard.edu/LEfSe) was performed to identify the significantly abundant taxa (phylum to genera) of bacteria among the different groups (LDA score > 2, *P* < 0.05).

### Analysis of ecological surveillance data

Raw mosquito surveillance data were organized, analyzed, and visualized using WPS Office. For spatial stratification, six central districts (Gaoxin, Wuhou, Chenghua, Jinjiang, Qingyang, Jinniu) were classified as the central urban area, characterized by high population density and urbanization levels. Eleven peripheral districts/counties (Dujiangyan, Pengzhou, Qingbaijiang, Jintang, Jianyang, Xinjin, Chongzhou, Dayi, Qionglai, Pujiang) were designated as the outer ring areas, dominated by mountainous and rural habitats. Regions between the central and outer rings (Wenjiang, Pidu, Xindu, Longquanyi, Shuangliu, Tianfu New Area) were categorized as the secondary ring areas, representing transitional ecological zones.

Statistical analyses were performed using SPSS 18.0. One-way ANOVA under a completely randomized design was applied to assess differences in mosquito density across years, months, and spatial strata. Pairwise comparisons of multiple sample means were conducted using the Bonferroni correction, with statistical significance set at *P* < 0.05.

## Results

### Mosquito density surveillance results

**Fig 1:**
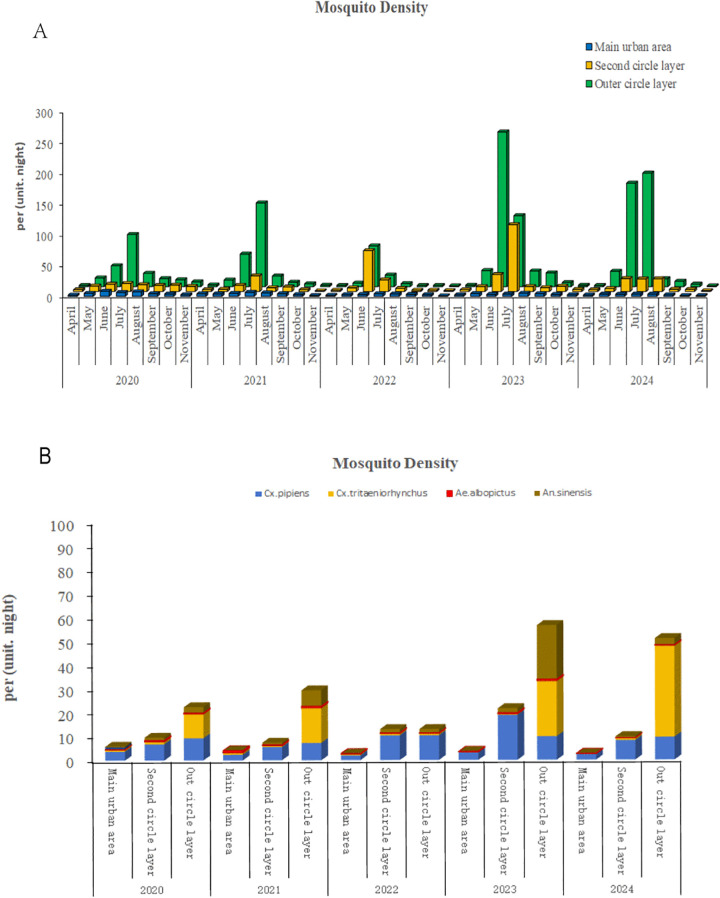
Mosquito density surveillance results. **(A)** Spatial variation in overall mosquito density across urban concentric rings. The outer ring areas exhibited significantly higher density compared to the secondary and central urban areas. **(B)** Seasonal mosquito density followed a unimodal pattern, peaking from June to July, with no significant interannual variation. Species composition varied spatially, with *Culex pipiens quinquefasciatus* dominating urbanized areas while *Culex tritaeniorhynchus and Anopheles sinensis* more abundant in rural outskirts. Key species are denoted as follows: *Cx.pipiens*: *Culex pipiens quinquefasciatus*; *Cx.tritaeniorhynchus*: *Culex tritaeniorhynchus*; *An. sinensis*: *Anopheles sinensis*; *Ae. albopictus*: *Aedes albopictus*.

From 2020 to 2024, a total of 9,581 mosquito traps were deployed in Chengdu, which collected 195,967 mosquitoes. The density indices (mosquitoes per trap per night) for key species were as follows: *Culex pipiens quinquefasciatus* (7.41), *Culex tritaeniorhynchus* (8.83), *Anopheles sinensis* (3.80), *Aedes albopictus* (0.41), and *Aedes aegypti* (0). The annual average mosquito densities from 2020 to 2024 were 13.56, 16.25, 9.32, 34.86, and 27.87 (mosquitoes per trap per night), respectively, with no statistically significant interannual differences (*F* = 1.366, *P* = 0.266). Seasonal trends exhibited a unimodal distribution, with a peak in June (99.67) and July (87.10). Monthly variations in density were statistically significant (*F* = 4.153, *P* = 0.002). Spatially, mosquito density varied significantly across regions (*F* = 8.354, *P* < 0.001). As shown in Fig 1A, the outer ring areas showed the highest density (34.69 mosquitoes per trap per night), followed by secondary ring areas (12.29), and the central urban areas had the lowest density (3.60). Species-specific spatial patterns were observed: *Culex pipiens quinquefasciatus* predominated in urbanized regions (primary and secondary rings), while *Culex tritaeniorhynchus* and *Anopheles sinensis* dominated in rural outer ring areas. Additionally, with the exception of 2022, the density of *Aedes albopictus* was consistently and significantly lower in the central urban areas than in both the secondary and outer rings. The detailed spatial and temporal distributions are presented in Fig 1B, with comprehensive data provided in S1–S5 Tables.

### Sequencing results and ASV classification

**Fig 2:**
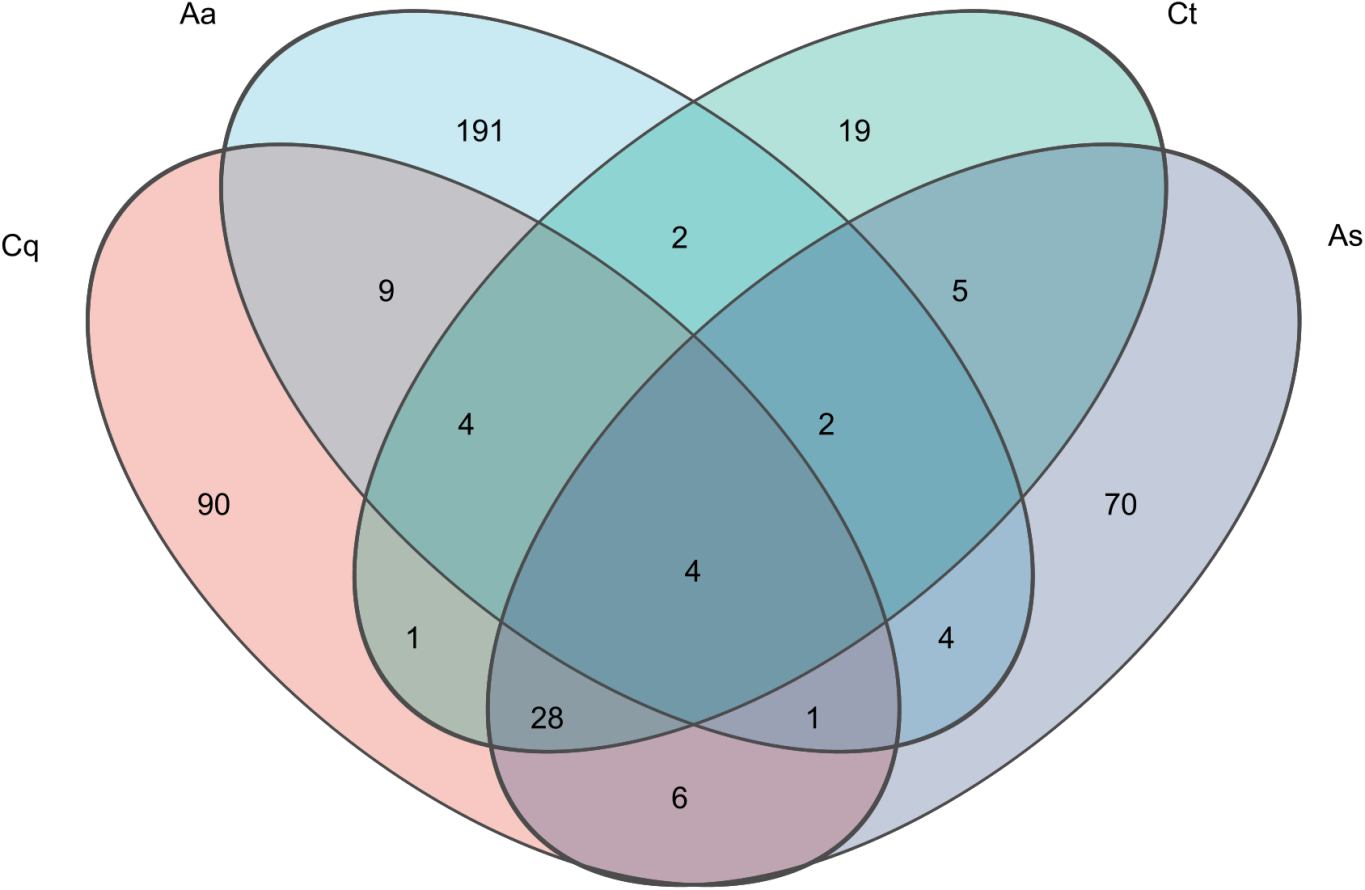
Venn diagram of symbiotic bacteria. Venn diagram analysis reveals minimal shared microbiota, with only four bacterial species common to all four host species. *Aedes albopictus* possesses the highest number of unique symbionts (191), contrasting sharply with *Culex tritaeniorhynchus* (19 unique species). Key species are abbreviated as follows: Cq: *Culex pipiens quinquefasciatus*; Aa: *Aedes albopictus*; Ct: *Culex tritaeniorhynchus*; As: *Aedes pullatus*.

A total of 297,649 high-quality sequences were obtained after quality control, with an average sequence length of 1,490 bp (range: 1,111–1,782 bp). Sequences were clustered at 100% similarity and rarefied to the minimum sequence number(4,090) to standardize sequencing depth. Taxonomic annotation of Amplicon Sequence Variants (ASVs) was performed using QIIME2, yielding 717 ASVs corresponding to 436 bacterial species. Venn diagram analysis of ASV abundance revealed distinct microbial community structures among mosquito species (Fig 2). *Aedes albopictus* exhibited the highest number of unique symbiotic bacterial species (191), while *Culex tritaeniorhynchus* harbored the fewest unique species (19). Only four bacterial species were shared across all four mosquito species.

### Alpha diversity analysis

**Fig 3:**
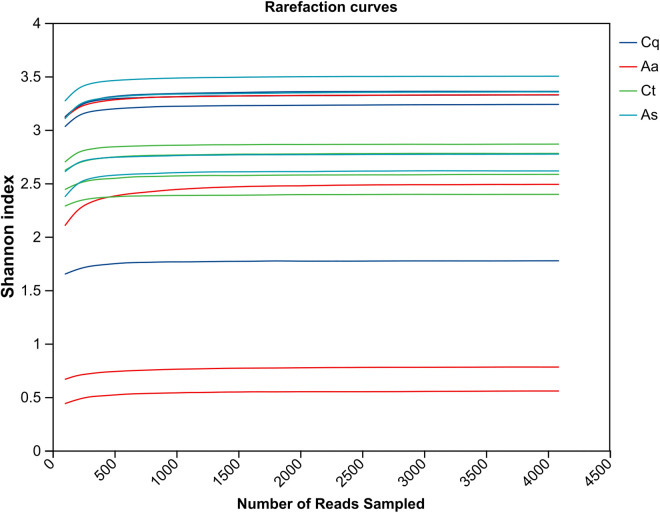
Rarefaction curvesin different mosquito species. Shannon index curves plateau beyond 500 sequences per sample, indicating sufficient sequencing depth to capture microbial diversity.

**Fig 4:**
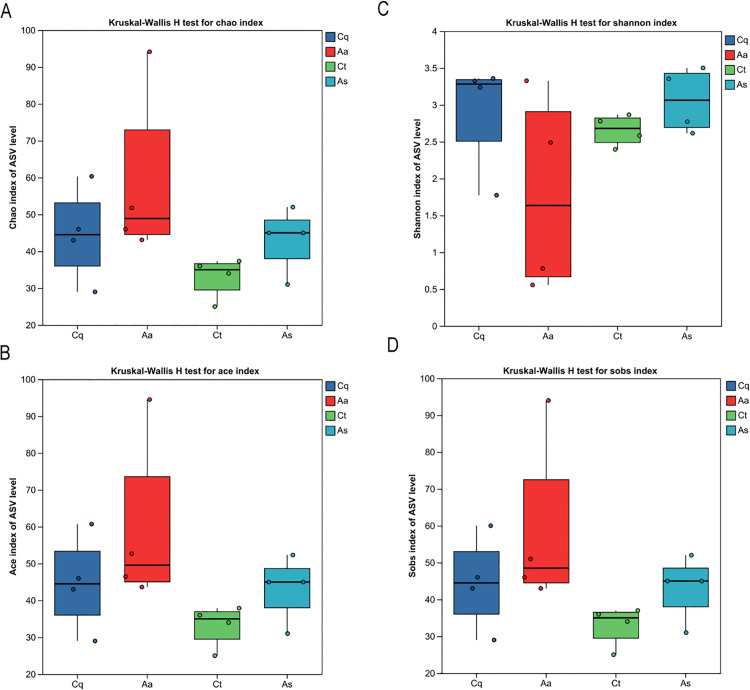
Distribution of Alpha diversity index boxplot. **(A)** Chao1 index, **(B)** Ace index, **(C)** Shannon index, and **(D)** Observed species (Sobs). *Aedes albopictus* (Aa) exhibited the highest species richness, while *Armigeres subalbatus* (As) showed the highest community diversity. No significant differences were detected among species following multiple-test correction (Wilcoxon rank-sum test, Padj > 0.05).

Rarefaction curves (Fig 3) were generated by randomly subsampling sequencing data to assess sequencing depth saturation. When subsampling exceeded 500 sequences, Shannon index curves for all samples plateaued, indicating sufficient sequencing depth to capture the majority of microbial species. Alpha diversity indices (Simpson, Shannon, Chao1, and coverage) were statistically analyzed to evaluate microbial diversity and species richness (Fig 4). Chao and ACE indices exhibited positive correlations with total community richness, while the Shannon index positively correlated with diversity and the Simpson index inversely correlated with diversity. The *Aedes albopictus* group (Aa) displayed the highest mean values for ACE (59.32 ± 23.76), Chao1 (58.75 ± 23.87), and Sobs (58.5 ± 23.90), whereas the *Culex tritaeniorhynchus* group (Ct) exhibited the lowest values (ACE: 33.22 ± 5.71; Chao: 33.08 ± 5.56; Sobs: 33 ± 5.48). The *Armigeres subalbatus* group (As) demonstrated the highest Shannon index (3.06 ± 0.43) and the lowest Simpson index (0.098 ± 0.082). Wilcoxon rank-sum tests revealed no statistically significant differences in richness, diversity, or evenness among mosquito species after multiple-test correction (Padjust > 0.05).

### Beta diversity analysis

**Fig 5:**
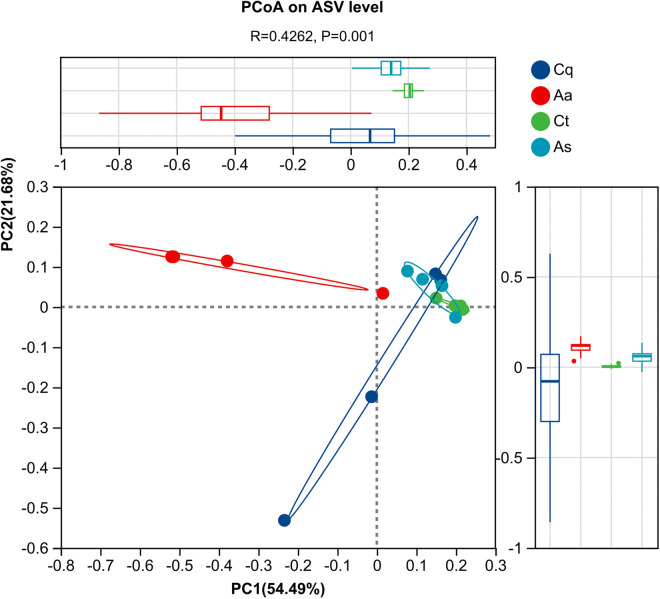
Principal Coordinates Analysis (PCoA) based on Weighted UniFrac distance. Principal Coordinate Analysis (PCoA) based on Weighted UniFrac distances shows clear separation among species. *Aedes albopictus* (Aa) forms a distinct cluster, while *Culex tritaeniorhynchus* (Ct) and *Armigeres subalbatus* (As) exhibit partial overlap. Ellipses represent 95% confidence intervals. ANOSIM confirms significant moderate differentiation between species communities (*R* = 0.43, *P* = 0.001).

Beta diversity was assessed using Weighted UniFrac distances and visualized via Principal Coordinate Analysis (PCoA) with ANOSIM (999 permutations). The PCoA plot (Fig 5) illustrated distinct clustering patterns among mosquito species, where proximity along the X- and Y-axes (relative distance units) indicated higher microbial community similarity. Ellipses represent 95% confidence intervals. *Culex tritaeniorhynchus* (Ct) and *Armigeres subalbatus* (As) exhibited partial overlap, while *Aedes albopictus* (Aa) remained distinctly separated from other groups. ANOSIM yielded an R-value of 0.43 (*P* = 0.001), indicating moderate differentiation (0.25 ≤ *R* < 0.5) in symbiotic microbiota composition among species.

### UPGMA clustering analysis

**Fig 6:**
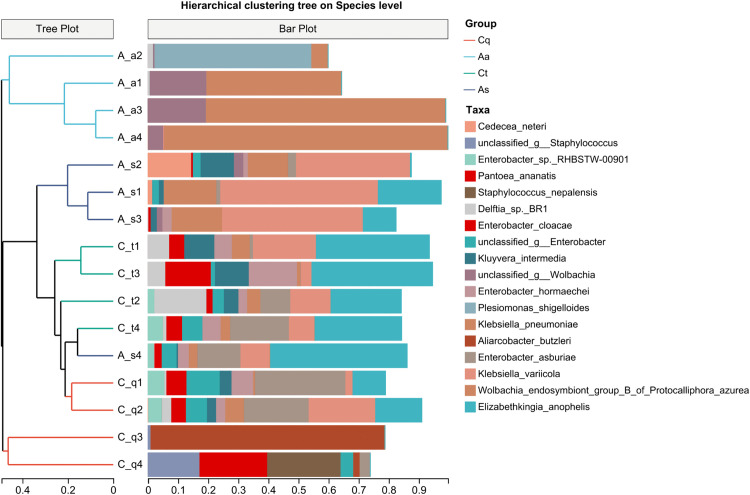
Hierarchical clustering analysis of mosquito symbiotic microbiota at the species level. The UPGMA dendrogram, based on Bray-Curtis dissimilarity, illustrates the relationships among microbial communities. *Ae. albopictus* (Aa) forms a distinct cluster, while the two Culex species (*Cx. pipiens*, Cq and *Cx. tritaeniorhynchus*, Ct) cluster together, indicating genus-level similarity in their microbiota. Notably, one subgroup of *Ar. subalbatus* (A_s4) clusters with Culex tritaeniorhynchus (C_t3), a pattern consistent with the PCoA results.

Hierarchical clustering (UPGMA algorithm) and Bray-Curtis distance-based heatmap analysis (Fig 6) quantified interspecific microbial abundance differences. Branch lengths in the dendrogram reflected the degree of dissimilarity, while heatmap color gradients (red = greater divergence) highlighted sample dissimilarity. *Aedes albopictus* (Aa) formed a distinct clade, whereas *Culex pipiens quinquefasciatus quinquefasciatus* (Cp) and *Culex tritaeniorhynchus* (Ct) clustered closely, suggesting a shared microbial community structure at the genus level. Notably, one *Armigeres subalbatus* subgroup (A_s4) exhibited proximity to *Culex tritaeniorhynchus* (C_t3), diverging from conspecific subgroups, aligning with PCoA results.

### Microbial community structure and differential analysis

**Fig 7:**
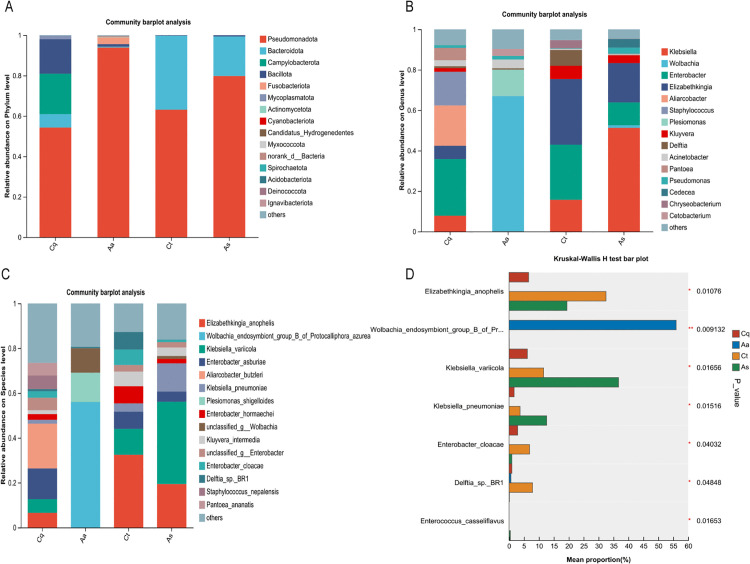
Differential analysis of bacterial relative abundance across taxonomic levels. **(A)** Phylum-level distribution of bacterial communities shows *Pseudomonadota* as the dominant phylum across all species, with varying abundance among host species. **(B)** Genus-level composition reveals host-specific colonization patterns, including *Wolbachia dominance* in *Ae. albopictus* and *Klebsiella prevalence* in *Ar. subalbatus*. **(C)** Species-level profile highlights dominant symbiotic species with distinct host associations. **(D)** Differential abundance analysis identifies significantly enriched bacterial species across host species (Kruskal-Wallis test with FDR correction; **0.001 < *P* ≤ 0.01; *0.01 < *P* ≤ 0.05).

Taxonomic annotation identified 23 phyla, 38 classes, 60 orders, 84 families, 116 genera, and 179 species. Dominant phyla (Fig 7A) included *Pseudomonadota* (relative abundance: 54.27% in Cx. Pipiens, 93.89% in *Ae. albopictus*, 63.15% in *Cx. tritaeniorhynchus*, 79.81% in *Ar. subalbatus*), *Bacteroidota* (36.57% in *Cx. tritaeniorhynchus*, 19.59% in *Ar. subalbatus*), *Campylobacterota* (19.96% in Cx. pipiens), and *Fusobacteriota* (3.37% in *Ae. albopictus*). At the genus level (Fig 7B), *Enterobacter* (28.03%), *Aliarcobacter* (19.96%), and *Staphylococcus* (16.66%) dominated Cx. pipiens; *Wolbachia* (66.90%), *Plesiomonas* (13.03%), and *Acinetobacter* (4.36%) prevailed in *Ae. albopictus*; *Elizabethkingia* (32.46%), *Enterobacter* (27.24%), and *Klebsiella* (15.70%) were prominent in *Cx. tritaeniorhynchus*; and *Klebsiella* (51.27%), *Elizabethkingia* (19.43%), and *Enterobacter* (19.43%) dominated *Ar. subalbatus*. Species-level analysis revealed *Aliarcobacter butzleri* (19.96%, Cx. pipiens), *Wolbachia endosymbiont group B* of *Protocalliphora azurea* (55.98%, *Ae. albopictus*), *Elizabethkingia anophelis* (32.46%, *Cx. tritaeniorhynchus*), and *Klebsiella variicola*(36.67%, *Ar. subalbatus*) as dominant species.

At the species level, interspecies differences in bacterial abundance among mosquito groups were analyzed using the Kruskal-Wallis rank-sum test, with results adjusted for multiple comparisons via the false discovery rate (FDR) method. Statistical analyses revealed significant species-specific enrichment patterns for several bacterial species with public health relevance. The relative abundances of these key species across the four mosquito species are detailed below (Fig 7C).

Statistical analysis of species abundance using one-way ANOVA revealed significant interspecific differences across the four mosquito species (Fig 7D). *Wolbachia endosymbiont group B* was overwhelmingly dominant in *Ae. albopictus* (55.98%), with only trace presence in *Cx. tritaeniorhynchus* (0.024%) and *Ar. subalbatus* (0.043%), and complete absence in *Cx. pipiens* (*P* = 0.009) **.

*Elizabethkingia anophelis* was primarily associated with *Cx. tritaeniorhynchus* (32.46%), exhibiting significantly higher abundance than in *Ar. subalbatus* (19.39%), *Cx. pipiens* (6.54%), or *Ae. albopictus* (0%) (*P* = 0.011) *.

The genus *Klebsiella* showed distinct partitioning: *K. variicola* was enriched in Ar. subalbatus (36.67%) compared to other species (*P* = 0.016) *, and *K. pneumoniae* likewise reached its highest abundance in *Ar. subalbatus* (12.6%; *P* = 0.015)*.

Two species, *Enterobacter cloacae* (6.86%; *P* = 0.04) * and *Delftia sp. BR1* (7.86%; *P* = 0.048) *, were significantly enriched in *Cx. tritaeniorhynchus*. Enterococcus casseliflavus was exclusively detected in *Ar. subalbatus* (0.44%).

### Microbial species abundance clustering analysis

At the genus level taxonomic annotation and abundance profiling were conducted across two dimensions: mosquito species groups and individual samples. A heatmap was generated with the horizontal axis representing sample or group labels, the vertical axis listing microbial species names, and a phylogenetic clustering tree at the family level displayed on the left. Color gradients in the heatmap visually represented species distribution across samples, with warmer hues indicating higher abundance values.

**Fig 8:**
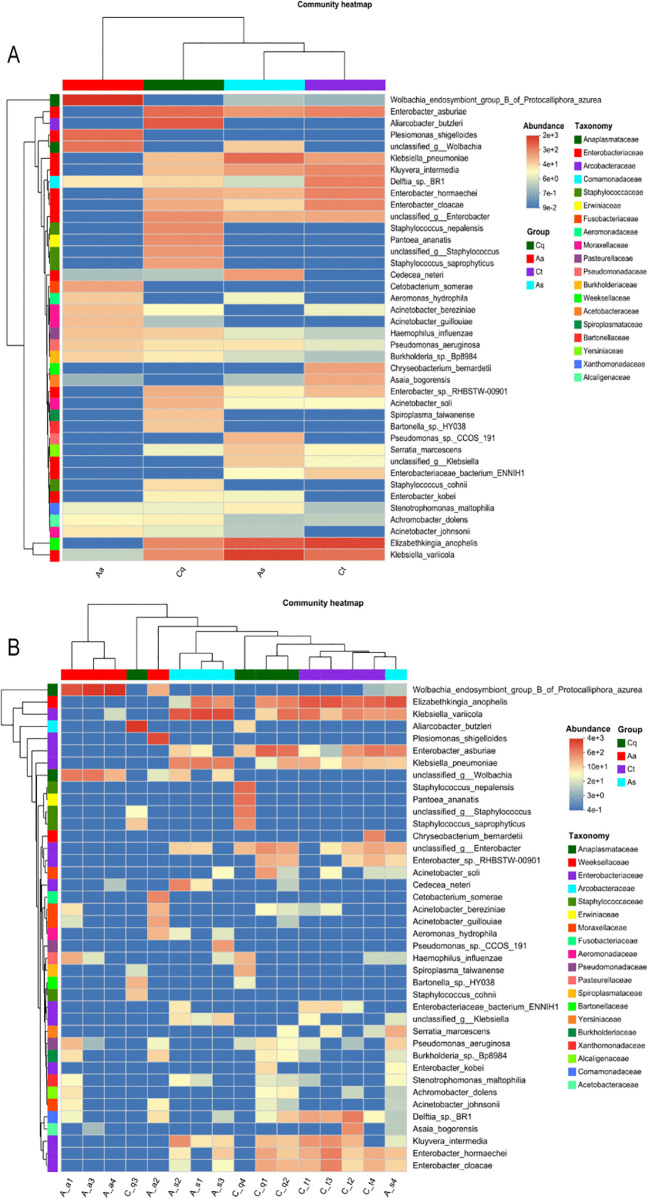
Species abundance clustering heatmap. **(A)** Distinct clustering patterns of symbiotic bacteria among the four mosquito species, with color gradients indicating abundance levels (warmer hues represent higher values). The phylogenetic tree on the left displays evolutionary relationships at the family level. *Cx. tritaeniorhynchus* exhibits the highest number of high-abundance taxa (n = 7), while *Ae. albopictus* and *Ar. subalbatus* show fewer dominant taxa (n = 3 each). **(B)** Average linkage clustering of individual samples reveals moderate variations in microbial abundance among conspecific subgroups, demonstrating intra-species heterogeneity in microbiota composition.

As illustrated in Fig 8A, distinct clustering patterns of symbiotic bacteria were observed among the four mosquito species. *Aedes albopictus* and *Armigeres subalbatus* each contained three taxa with relatively high abundance (values > 1,000), whereas *Culex pipiens quinquefasciatus* and *Culex tritaeniorhynchus* exhibited four and seven high-abundance taxa, respectively. Average linkage clustering of individual samples (Fig 8B) further demonstrated moderate variations in microbial abundance among subgroups within the same mosquito species.

### Phylogenetic analysis of bacterial species

**Fig 9:**
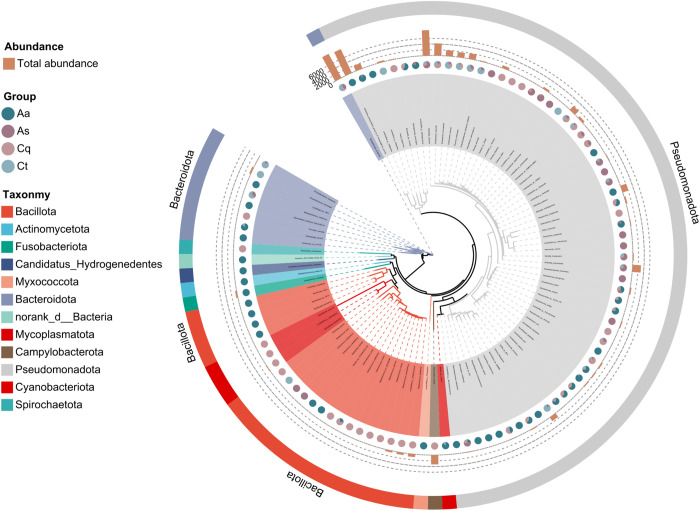
Species-level phylogenetic tree. The tree illustrates distinct phylum-level distribution patterns, with *Pseudomonadota* ubiquitously dominating across all hosts and *Bacteroidota* showing species-specific enrichment. Unclassified taxa (e.g., *norank_f__Enterobacteriaceae*) cluster within core clades of established phyla, indicating close phylogenetic affinity to known genera. Notably, the candidate phylum *Candidatus Hydrogenedentes* forms a basal branch exclusively in *Ae. albopictus*, demonstrating close evolutionary relationship with mosquito-associated spirochetes and suggesting presence of novel functional lineages. **Note:** Pie chart colors indicate corresponding phyla. Branches represent distinct bacterial species. Stacked bar charts and proportional pie charts illustrate the abundance distribution of these bacteria across different mosquito species.

A maximum likelihood phylogenetic tree was constructed based on all Amplicon Sequence Variants (ASVs) using representative sequences annotated against the SILVA database (Fig 9). At the phylum level, *Pseudomonadota* was ubiquitously distributed across all four mosquito species, dominating in *Aedes albopictus* and *Culex tritaeniorhynchus*. In contrast, *Bacteroidota* exhibited preferential enrichment in *Armigeres subalbatus* and *Culex pipiens quinquefasciatus*, likely associated with species-specific ecological niches or physiological requirements. Unclassified taxa, such as *norank_f__Enterobacteriaceae* and *norank_p__Bacteroidota*, clustered within core evolutionary clades of *Pseudomonadota* and *Bacteroidota*, respectively, despite lacking genus-level classification. These taxa showed high phylogenetic affinity to known genera (*Kluyvera* and *Sphingobacterium*), suggesting their potential membership within these taxonomic groups. Notably, *norank_p__Candidatus_Hydrogenedentes*, the sole representative of the candidate phylum *Hydrogenedentes*, formed a basal branch in the tree and clustered with *norank_d__Bacteria*. Both taxa, which were exclusively detected in the *Ae. albopictus* group, displayed close phylogenetic proximity to *Entomospira nematocera*, a recently identified mosquito-associated spirochete, indicating the presence of undercharacterized functional microbiota within this lineage.

### Functional prediction of microbial communities

**Fig 10:**
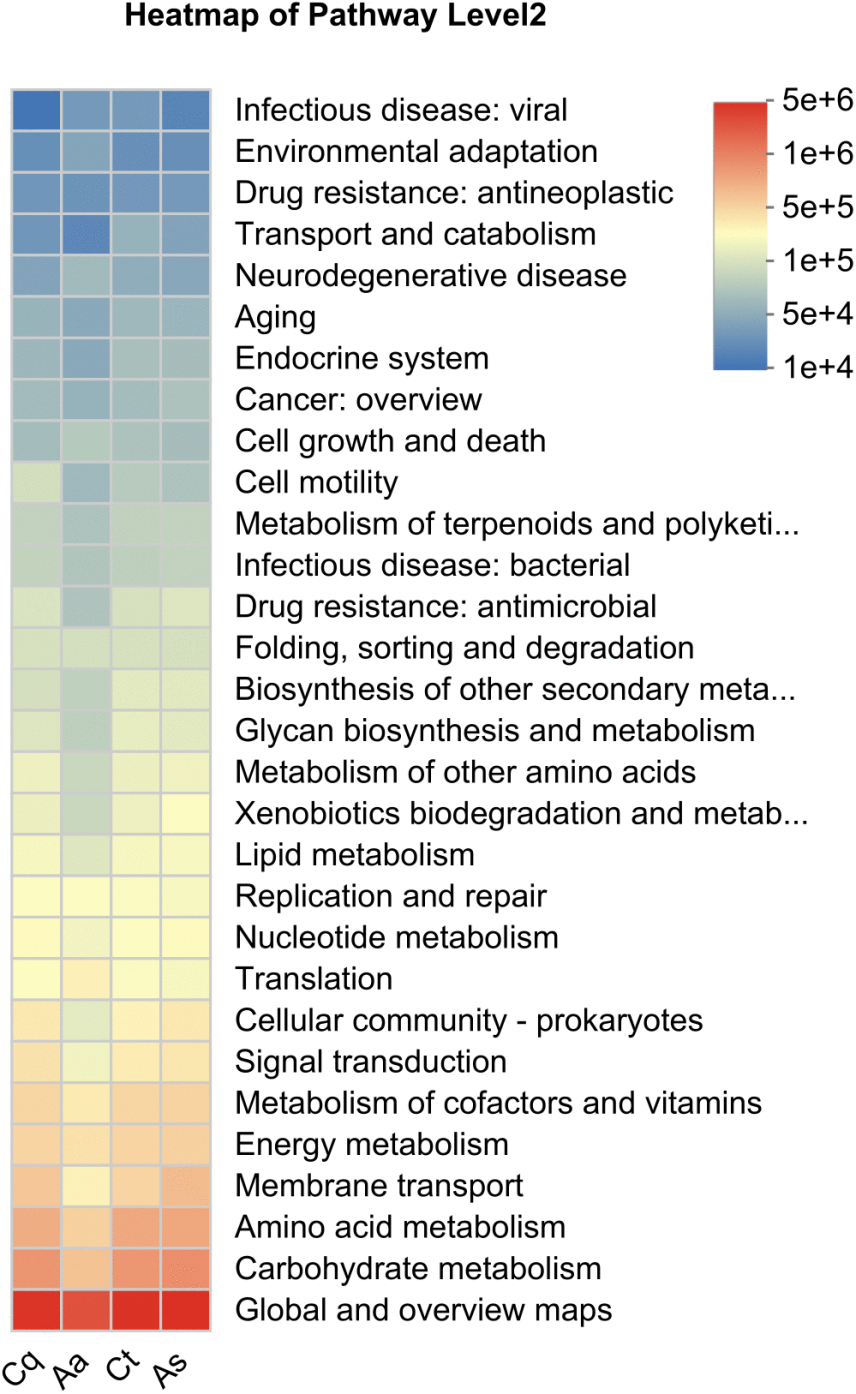
KEGG functional abundance prediction analysis heatmap. The relative abundance of each pathway is represented by color intensity. Kruskal-Wallis H tests revealed significant among the mosquito species in the “Human Diseases” pathway and several of its subcategories.

Functional annotation of metabolic pathways in mosquito-associated symbiotic bacteria was performed using PICRUSt2 (v2.2.0-b) based on the Kyoto Encyclopedia of Genes and Genomes (KEGG). A total of 334 functional pathways were identified, and a heatmap was generated to visualize the top 30 most abundant pathways (Fig 10). The heatmap displays functional abundance distributions across mosquito species using color gradients, where the horizontal axis represents group labels, the vertical axis lists pathway names, and color intensity is proportional to abundance.

As shown in Fig 10, significant differences in pathway abundances were observed among groups. Statistical analysis using the Kruskal–Wallis H test (*P* ＜ 0.05) identified significant differences in the Level 1 pathway “Human Diseases” (*P* = 0.049). Subsequent analysis of its Level 2 sub-pathways showed significant variations in “Endocrine and metabolic disease” (*P* = 0.015), “Infectious disease: bacterial” (*P* = 0.045), “Infectious disease: parasitic” (*P* = 0.009), “Infectious disease: viral” (*P* = 0.042).

Notably, *Aedes albopictus* exhibited significantly higher abundances in “Endocrine and metabolic disease” relative to the other three species. For “Infectious disease: viral”, pathway abundances followed *Culex tritaeniorhynchus* > *Aedes albopictus* > *Armigeres subalbatus* > *Culex pipiens quinquefasciatus quinquefasciatus*. In contrast, *Aedes albopictus* showed the lowest abundances in both “Infectious disease: bacterial” and “Infectious disease: parasitic”, which were highest in *Culex pipiens quinquefasciatus* and *Culex tritaeniorhynchus*, respectively.

## Discussion

Mosquitoes pose a significant global health burden by transmitting pathogens responsible for diseases such as Japanese encephalitis, malaria, and dengue fever, threatening approximately 3.9 billion people across 120 countries [13]. Chengdu, a subtropical city in southwestern China, provides an ideal habitat for mosquito proliferation due to its climate and ongoing urbanization. As a hub for tourism, trade, and international events, Chengdu faces heightened risks of emerging or re-emerging mosquito-borne diseases. Historical surveillance data (2013–2017) identified *Culex pipiens quinquefasciatus* (50.43%), *Culex tritaeniorhynchus* (35.85%), and A*rmigeres subalbatus* (7.39%) as the dominant mosquito species in Chengdu. Our analysis of 2020–2024 surveillance data revealed no significant interannual differences in overall mosquito density. However, spatial stratification showed the highest density in outer ring areas (34.69 mosquitoes per trap per night), followed by secondary ring areas (12.29), and the lowest in central urban zones (3.60). This spatial pattern likely reflects variations in vegetation cover, ecological diversity, human mobility, and public health intervention efficacy. Notably, the density of *Aedes albopictus* was also found to be significantly lower in the urban core compared to the peripheral rings in all years except 2022. As a semi-domestic species with peak activity at dawn and dusk, *Aedes albopictus* is less effectively captured by standard light traps. This sampling bias, combined with factors such as intense artificial lighting and altered human activity patterns in the urban core, may collectively explain its relatively low density in central areas. Furthermore, the rural predominance of *Culex tritaeniorhynchus* and *Anopheles sinensis* aligns with previous studies, reinforcing the link between species distribution and land use.

Our sequencing results revealed striking interspecies differences in symbiotic microbiota composition. *Aedes albopictus* harbored the highest number of unique bacterial species (191), while only four species were shared across all four mosquito groups. Although alpha diversity metrics (richness, Shannon index) showed numerical variations among species, no statistically significant differences were observed. In contrast, beta diversity analyses (UPGMA, PCoA) demonstrated distinct clustering patterns: *Culex tritaeniorhynchus* and *Culex pipiens quinquefasciatus* exhibited closer phylogenetic proximity, with partial overlap to *Armigeres subalbatus*, whereas *Aedes albopictus* formed a separate cluster. These findings align with prior studies highlighting niche-driven microbial divergence in mosquitoes [14]. At the phylum level, *Pseudomonadota* dominated all species (54.27–93.89%), while secondary dominant phyla varied (*Bacteroidota* in *Culex tritaeniorhynchus*, *Campylobacterota* in *Culex pipiens quinquefasciatus*, and *Fusobacteriota* in *Aedes albopictus*), consistent with global mosquito microbiome trends [15,16]. The phylum *Pseudomonadota* occupies a significant position in the gut microbiota of insects. Specifically, *S. ureilytica* has been demonstrated to directly target and eliminate Plasmodium parasites through the secretion of a lipase protein, AmLip. Conversely, proteins secreted by Serratia marcescens have been identified as major effectors that heighten mosquito susceptibility to arboviral infections, thereby enhancing the transmissibility of dengue virus. Investigations into the impact of CHIKV infection on the overall bacterial community in Aedes mosquitoes revealed a positive correlation between the abundance of *Enterobacteriaceae* (within this phylum) and CHIKV infection status, suggesting a potential cooperative interaction between these bacteria and the virus. Collectively, these findings indicate that bacteria within *Pseudomonadota* may play critical roles in the transmission of mosquito-borne diseases [17,18].

*Bacteroidota* is renowned for its capacity to degrade complex polysaccharides (McKee). Furthermore, certain members of this phylum have been shown to modulate host immune responses through pathways involving peroxisomes, autophagy, PI3K-Akt signaling, and lysozyme activity (Huber). These observations collectively underscore the role of *Bacteroidota* as a central hub within the complex network of environment–host–pathogen interactions [19,20]. *Campylobacterota* has been identified in diverse environments, as well as in the digestive tracts of humans, other mammals, and aquatic invertebrates such as sea urchins, shrimp, and scallops. Notable pathogenic members, including *Helicobacter pylori* and *Campylobacter jejuni*, are extensively studied as common agents of gastrointestinal diseases. Meanwhile, certain autotrophic representatives have been demonstrated to serve as nutritional supplements that effectively promote host development. Currently, there is no definitive evidence indicating that *Campylobacterota* influences immune mechanisms in insects in response to pathogenic infections [21,22].

Genus- and species-level analyses further identified pathogenic or opportunistic taxa (e.g., *Enterobacter*, *Campylobacter*, *Staphylococcus*), underscoring their potential role in human infections under specific conditions [23–25].

Notably, species-specific abundance patterns were observed for key bacteria with public health implications. *Wolbachia (clade B)*, exclusively detected in *Ae. albopictus,* has been shown to suppress dengue virus replication in *Aedes aegypti* by enhancing host immune responses [26] and reducing West Nile virus titers in cell lines [27]. Field-collected *Aedes aegypti* in Chengdu exhibited dual infections of *Wolbachia wAlbB* and an unclassified strain, diverging from the common *wAlbA/wAlbB* co-infections reported in Henan, and Nanjing [28,29]. This unique infection pattern warrants further investigation into its mechanistic and epidemiological implications. Similarly, *Elizabethkingia anophelis*, enriched in *Culex tritaeniorhynchus*, has demonstrated inhibitory effects on Zika virus infection in *Aedes aegypti* [30] and *Plasmodium* development in *Anopheles gambiae* [31], suggesting its potential utility in biocontrol strategies.

Of particular concern is the high abundance of *Klebsiella variicola* and *Klebsiella pneumoniae* in *Armigeres subalbatus. K. variicola*, an emerging opportunistic pathogen, is associated with neonatal sepsis, pneumonia, and bloodstream infections (BSI), often exhibiting higher mortality rates than *K. pneumoniae* [32]. Although direct evidence of mosquito-to-human transmission remains lacking, the prevalence of these pathogens in mosquitoes highlights a potential public health risk.

Furthermore, phylogenetic analysis revealed that unclassified taxa (*norank_d__Bacteria* and *norank_p__Candidatus_Hydrogenedentes*) formed a distinct cluster. Both of these taxa were exclusively detected in the *Aedes aegypti* group and exhibited close phylogenetic affiliation with *Entomospira nematocera,* a mosquito-associated spirochete originally isolated from Culex and Anopheles mosquitoes in the Czech Republic. This spirochete, along with *Entomospira culicis* and *Entomospira entomophilus* identified in the same study, was only recently established as novel species in 2020 [33]. The genus *Entomospira* shows a close phylogenetic relationship with anaerobic spirochetes of marine sediment origin, such as *Oceanispirochaeta* and *Sediminispirochaeta*, suggesting a potential anaerobic or microaerophilic lifestyle. However, their ecological functions and interactions with the host remain largely unexplored. The unclassified lineages identified in our study may represent hitherto unrecognized functional microbiota. To elucidate their evolutionary origins and ecological roles in mosquito biology, future studies employing targeted approaches, such as microbial isolation or metagenomics, are warranted.

The unclassified taxa *norank_f__Enterobacteriaceae* and *norank_p__Bacteroidota* clustered within the core evolutionary clades of *Pseudomonadota* and *Bacteroidota*, respectively, and exhibited high phylogenetic affinity to the established genera *Kluyvera* and *Sphingobacterium*. This suggests their potential membership as yet-undescribed representatives of these taxonomic groups.

*Kluyvera*, a Gram-negative bacterium, possesses both cellulose-degrading and nitrogen-fixing capabilities, potentially facilitating host amino acid synthesis by providing nitrogen-containing compounds [34,35].Although studies on the interaction between *Sphingobacterium* and insects remain relatively limited, functional insights have been well documented in closely related genera within the same family. For instance, *Sphingomonas* [36] has been shown to enhance resistance in cotton bollworm (Helicoverpa armigera) by degrading imidacloprid, while *Novosphingobium* [37] can synergize with other gut microbes to promote larval development.However, the ecological functions of these bacteria and their mechanisms of interaction with mosquito hosts remain largely unexplored.

Numerous studies have confirmed that spatial scales—such as microenvironments, land use patterns, and geographic extent—exert profound and complex influences on regional microbial communities through multiple pathways. For instance, research on insects of the genus Jalysus across the United States revealed significant divergence in Burkholderia strains among geographically distinct populations, with geographic location accounting for 27% of the observed strain variation [38]. Similarly, comparative analyses of mosquito microbiota from Spanish and São Tomé populations demonstrated that geographical isolation shapes symbiotic bacterial composition [39]. These patterns are likely mediated by a combination of environmental factors, including chemical stressors, temperature, precipitation, and resource availability. In a controlled rearing experiment, Laura E. Brettell et al. further demonstrated that even subtle inconsistencies in rearing conditions can alter the microbial profile of *Aedes aegypti* [40].

However, the present study did not explore the spatial variation in mosquito symbiotic bacteria across different geographical settings. This limitation constrains our ability to contextualize the observed microbial patterns within broader ecological and spatial frameworks. Future investigations should incorporate multi-scale sampling strategies encompassing diverse biogeographic regions, which would help disentangle the effects of geographic distance, environmental heterogeneity, and host dispersal on microbial assembly. Moreover, integrating metagenomic and environmental metadata would facilitate a mechanistic understanding of how specific abiotic and biotic factors—such as climate, vegetation, and land use—interact to shape mosquito-associated microbiomes in natural settings.

Functional annotation of pathway results revealed significant divergence in the functional potential of mosquito symbiotic microbiomes. Specifically, *Culex tritaeniorhynchus* and *Aedes albopictus* exhibited significant enrichment in the “Infectious Diseases: Viral” pathway, consistent with their recognized vector competence [41,42], suggesting their symbiotic bacterial communities may create a favorable environment for viral replication or transmission. In contrast, *Armigeres subalbatus* and *Culex pipiens quinquefasciatus quinquefasciatus* showed lower abundance of such pathways, indicating potentially weaker microbial support for viral propagation. Multiple previous studies have indicated that symbiotic bacteria may positively or negatively influence host pathogen response through metabolite production or immune regulation [43,44]. The unique enrichment of parasite disease-related pathways within the symbiotic bacterial community of *Culex tritaeniorhynchus* indicates a potential ecological niche for parasite transmission. Although not established as a known parasite vector, this abundance divergence suggests its symbiotic microbiota may influence host susceptibility to pathogenic parasites by constructing a microbial environment capable of differential interactions with pathogens. It is crucial to note that these functional predictions were derived from PICRUSt2, which infers metabolic potential from 16S rRNA data. Thus, while these findings highlight intriguing ecological patterns, future studies should integrate metagenomic sequencing and experimental validation to confirm these functional attributes and their causal roles in pathogen transmission.

In summary, the species-specific metabolic signatures identified in mosquito symbiotic bacteria may contribute to their differential vector competencies. Manipulating these microbial communities presents a promising strategy for disrupting pathogen transmission.

## Conclusion

In conclusion, this study integrates five years of ecological surveillance with high-resolution PacBio full-length 16S rRNA sequencing to provide a comprehensive overview of the mosquito population dynamics and symbiotic microbiota in Chengdu, a subtropical megacity in China. Our findings reveal significant spatial stratification in mosquito density, with rural outer-ring areas sustaining the highest burden, dominated by *Culex tritaeniorhynchus* and *Anopheles sinensis*, while *Culex pipiens quinquefasciatus* was more adapted to urbanized settings. The low density of *Aedes albopictus* in central urban areas highlights the impact of urbanization and sampling methods on species-specific surveillance outcomes.

Microbiome analysis demonstrated strong host-species structuring of bacterial communities. *Aedes albopictus* harbored the most unique symbiotic bacteria, and beta diversity analysis confirmed distinct clustering of microbiota by host species, driven by the ubiquitous dominance of Pseudomonadota and variations in secondary phyla. Crucially, our functional prediction analysis revealed significant and species-specific disparities in the abundance of human disease-associated pathways among the mosquito symbiotic microbiota. Notably, *Culex tritaeniorhynchus* and *Aedes albopictus* showed enrichment in viral infectious disease pathways, aligning with their known roles as primary arbovirus vectors. In contrast, *Aedes albopictus* exhibited the lowest abundance in bacterial and parasitic infectious disease pathways, which were more prominent in *Culex pipiens quinquefasciatus* and *Culex tritaeniorhynchus*, respectively.

The species-specific enrichment of key bacteria with public health implications, such as Wolbachia in *Aedes albopictus*, Elizabethkingia in *Culex tritaeniorhynchus*, and pathogenic Klebsiella in *Armigeres subalbatus*, underscores the potential of these microbial associations to influence vector competence and disease risk. The discovery of unclassified taxa phylogenetically linked to novel mosquito-associated spirochetes further highlights the untapped diversity of the mosquito microbiome.

This work establishes the first foundational baseline of mosquito-microbe interactions in Chengdu, offering critical insights for public health. The identified host-specific microbial and functional signatures present actionable targets for developing novel biocontrol strategies, such as the precise leveraging of Wolbachia, and for enhancing early-warning surveillance systems for mosquito-borne pathogens. A key limitation of this study is the lack of spatial replication in microbial sampling, which constrains our ability to discern geographic influences on microbiome composition. Future research should incorporate multi-scale geographic sampling, metagenomic sequencing, and experimental validation to definitively establish the causal relationships between symbiotic microbiota and vector competence, ultimately informing more effective and targeted vector intervention measures in rapidly urbanizing ecosystems.

## Supporting information

S1 Table Mosquito surveillance data for 2020: density and species composition by month and environment type. Number of traps deployed, total mosquitoes captured, overall density (per trap-night), and monthly abundance of five key mosquito species (*Culex quinquefasciatus*, *Culex tritaeniorhynchus*, *Aedes albopictus*, *Aedes aegypti*, *Anopheles sinensis*) across different environmental types in 2020.
(XLSX)

S2 Table Mosquito surveillance data for 2021: density and species composition by month and environment type in Chengdu. Number of traps deployed, total mosquitoes captured, overall density (per trap-night), and monthly abundance of five key mosquito species (*Culex quinquefasciatus*, *Culex tritaeniorhynchus*, *Aedes albopictus*, *Aedes aegypti*, *Anopheles sinensis*) across different environmental types in 2021.
(XLSX)

S3 Table Mosquito surveillance data for 2022: density and species composition by month and environment type in Chengdu. Number of traps deployed, total mosquitoes captured, overall density (per trap-night), and monthly abundance of five key mosquito species (*Culex quinquefasciatus*, *Culex tritaeniorhynchus*, *Aedes albopictus*, *Aedes aegypti*, *Anopheles sinensis*) across different environmental types in 2022.
(XLSX)

S4 Table Mosquito surveillance data for 2023: density and species composition by month and environment type in Chengdu. Number of traps deployed, total mosquitoes captured, overall density (per trap-night), and monthly abundance of five key mosquito species (*Culex quinquefasciatus*, *Culex tritaeniorhynchus*, *Aedes albopictus*, *Aedes aegypti*, *Anopheles sinensis*) across different environmental types in 2023.
(XLSX)

S5 Table Mosquito surveillance data for 2024: density and species composition by month and environment type in Chengdu. Number of traps deployed, total mosquitoes captured, overall density (per trap-night), and monthly abundance of five key mosquito species (*Culex quinquefasciatus*, *Culex tritaeniorhynchus*, *Aedes albopictus*, *Aedes aegypti*, *Anopheles sinensis*) across different environmental types in 2024.
(XLSX)
